# Virus-induced gene silencing (VIGS) in *Cannabis sativa* L.

**DOI:** 10.1186/s13007-019-0542-5

**Published:** 2019-12-26

**Authors:** Julia Schachtsiek, Tajammul Hussain, Khadija Azzouhri, Oliver Kayser, Felix Stehle

**Affiliations:** Laboratory of Technical Biochemistry, Department of Biochemical and Chemical Engineering, TU Dortmund University, Dortmund, Germany

**Keywords:** Virus-induced gene silencing, *Cannabis sativa*, *Cotton leaf crumple virus* (*CLCrV*), Post-transcriptional gene silencing (PTGS)

## Abstract

**Background:**

The raised demand of cannabis as a medicinal plant in recent years led to an increased interest in understanding the biosynthetic routes of cannabis metabolites. Since there is no established protocol to generate stable gene knockouts in cannabis, the use of a virus-induced gene silencing (VIGS) method, resulting in a gene knockdown, to study gene functions is desirable.

**Results:**

For this, a computational approach was employed to analyze the *Cannabis sativa* L. transcriptomic and genomic resources. Reporter genes expected to give rise to easily scorable phenotypes upon silencing, i.e. the *phytoene desaturase* (*PDS*) and *magnesium chelatase subunit I* (*ChlI*), were identified in *C. sativa.* Subsequently, the targets of specific small interfering RNAs (siRNAs) and silencing fragments were predicted and tested in a post-transcriptional gene silencing (PTGS) approach*.* Here we show for the first time a gene knockdown in *C. sativa* using the *Cotton leaf crumple virus* (*CLCrV*) in a silencing vector system. Plants transiently transformed with the *Agrobacterium tumefaciens* strain AGL1, carrying the VIGS-vectors, showed the desired phenotypes, spotted bleaching of the leaves. The successful knockdown of the genes was additionally validated by quantitative PCR resulting in reduced expression of transcripts from 70 to 73% for *ChlI* and *PDS*, respectively. This is accompanied with the reduction of the chlorophyll a and carotenoid content, respectively. In summary, the data clearly demonstrate the potential for functional gene studies in cannabis using the *CLCrV*-based vector system.

**Conclusions:**

The applied VIGS-method can be used for reverse genetic studies in *C. sativa* to identify unknown gene functions. This will gain deeper inside into unknown biosynthetic routes and will help to close the gap between available genomic data and biochemical information of this important medicinal plant.

## Background

*Cannabis sativa* L. is currently undergoing a renaissance in the treatment of various disease symptoms [1]. Recently, in many western countries, cannabis was legalized for medicinal purposes. However, due to the long-term worldwide cultivation ban of the plant, the systematic characterization of cannabis and their metabolites was neglected. In the last years many genomic and transcriptomic data became available [2, 3] but the functional characterization of gene functions lags behind. Up to date, only a few gene functions are evaluated. One approach which could accelerate the functional characterization of cannabis genes is the development of a reliable transformation protocol, which is still challenging [4]. However, first successful hemp regeneration was recently described using novel synthetic cytokinin derivatives [5]. This would enable the use of the CRISPR–Cas9 system to produce loss-of-function mutants. One possibility to study gene functions without using stable transformation protocols represent RNAi and virus-induced gene silencing (VIGS) approaches inducing transient gene knockdowns. Both have emerged as the most simple and straightforward methods for reverse genetics to characterize functional target genes.

**Fig. 1:**
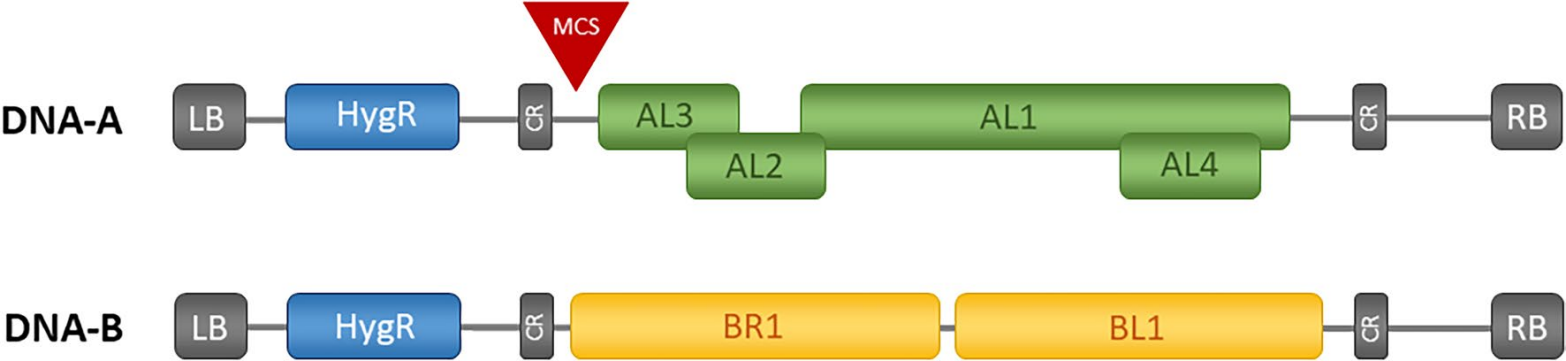
Schematic representation of *CLCrV****-***vectors T-DNA structure. In between the left and right border DNA-A or DNA-B are present in addition to a hygromycin resistance cassette (*HygR*). DNA-A of *CLCrV* consists of four genes (*AL1-AL4*) and DNA-B consists of two genes (*BR1* and *BL1*). The genes are flanked by the common regions (CR), consisting of the origin of replication

We utilized the *Cotton leaf crumple virus* (*CLCrV*) [6] for VIGS in *C. sativa*. The *CLCrV* is a ssDNA virus that belongs to the bipartite begomoviruses consisting of two 2.5 kb circular DNA molecules, named DNA-A and DNA-B (Fig. 1) [7]. The replication is facilitated by a so-called rolling circle mechanism in the nucleus, whereas the common region (~ 200 bp) upstream of the two bidirectional-promotors act as origin for the viral DNA replication. For replication, the host machinery is necessary, synthesizing single- and double-stranded DNA molecules [8, 9]. The viral DNA is mobile and is present as episomes that can move in and out of the nucleus but also between different cells, tissues, and organs. This leads to systemic infection of the plant [10, 11].

VIGS makes use of the innate plant defense system against virus infections [12, 13]. Begomoviruses transcribe their genomes in the nuclei [14]. Subsequently, dsRNA is synthesized by the RNA-dependent RNA polymerase (RDR) in the cytosol. The dsRNA then triggers post-transcriptional gene silencing (PTGS). This leads to the cleavage of the dsRNA and the generation of small interfering RNA (siRNA) molecules [15] that are recognized by the RNA-induced silencing complex (RISC) [16, 17]. Subsequently, the double-stranded siRNAs are melted into single-stranded ones and those are used by the RISC to screen for complementary sequences. The resulting dsRNA molecules i.e. complexes of siRNA and virus-ssRNA will then be degraded and the replication of the virus is slowed down. This results in an amplification of the siRNA enabling a transport throughout the plant. Therefore, silencing can be observed in distant cells and often in most parts of the plant [18]. VIGS exploits this RNA defense mechanism by mimicking a virus infection based on the delivery of artificial siRNA molecules to a plant cell. Thus, plant-specific mRNA molecules can be targeted resulting in a suppression of the gene expression and a reduction of the amount of the corresponding encoded proteins.

We aimed for a straightforward method to study the function of genes in *C. sativa.* Therefore, we established a VIGS system utilizing the *Cotton leaf crumple virus* (*CLCrV*) [6]. To visualize the knockdown, we used two maker genes resulting in a visible phenotype. First, the endogenous *phytoene desaturase* (*PDS*) gene was targeted. The *PDS* encodes an enzyme catalyzing the first step in the carotenoid biosynthetic pathway. The knockdown of the transcript will result in white leaves due to a photobleaching effect [19]. This is the result of negative feedback regulation, inhibiting the chlorophyll biosynthesis [20]. Second, the *magnesium chelatase subunit I* (*ChlI*) was selected. The magnesium chelatase (MgCh) is the primary enzyme for chlorophyll biosynthesis adding the magnesium ion into protoporphyrin IX. MgCh consists of three subunits called ChlH, ChlD and ChlI. Among them, ChlI is of significant importance in the chlorophyll biosynthesis. ChlI subunit interacts with ChlD to activate the ChlH, which thereby regulate the whole biosynthetic pathway [21, 22]. Therefore, a loss of function in ChlI inhibits chlorophyll biosynthesis resulting in a “yellow” phenotype.

Here, we successfully demonstrated the ability of VIGS using *CLCrV*. This enables to unravel gene functions, as well as to get in-depth insights to explore the distinguished biosynthetic pathway and development in this important medicinal plant.

## Methods

### Identification of candidate genes and the siRNAs

For the identification of the orthologues of *PDS* and *ChlI* in *C. sativa* a draft genome of *C. sativa* was downloaded from NCBI (GCA_000230575.2; [2]. Afterwards the standalone blast+ for Unix operating systems, provided by NCBI, was installed and configured (https://www.ncbi.nlm.nih.gov/books/NBK52640/). A database was created with the use of BLASTDB from in-house transcriptomic resources (not published) and the draft genome. The generated database (detailed methodology of standalone blast described by Hussain et al. [23], was used to search for homologous sequences of *PDS* and *ChlI* of *C. sativa* with blastn.

For the prediction of siRNAs from the identified candidate genes the publicly available bioinformatics tool pssRNAit (https://plantgrn.noble.org/pssRNAit/) was used with criteria described by Xu [24]. With this information silencing gene fragments of 300 to 400 base pairs with a GC content of 30% to 70% of *PDS* and *ChlI* were defined containing the predicted siRNA target sequences. For this, fragments in the 5′ untranslated regions (UTRs), 3′ UTRs and first 100 bp of the coding sequence were excluded.

### Identification of cannabis reference genes

For the identification of reference genes, suitable for quantitative real time PCR in *C. sativa*, the coding sequences from *Arabidopsis thaliana* L. of typically used reference genes *actin* (NM_001338359.1), *tubulin* (NM_106228)*, GAPDH* (NM_101214.4), *EF1a* (NM_125432.4)*, ubq10* (NM_178968.5)*, eIFa* (NM_104305.4)*, 18S rRNA* (X16077.1), *ubq5* (NM_116090.3) and *yls8* (AB047811.1) were downloaded from NCBI. To find the homologous sequences in *C. sativa* “blastn” 2.6.0+ was used. Sequences with a query coverage of more than 80%, an e-value of 1e10-3 and a homology greater than 70%, were chosen for a “blastx” analysis against the NCBI non-redundant (nr) database. Gene sequences with high homology to the respective tested gene sequences were declared as homologous sequences and can be found in Additional file 1.

### Evaluation of reference genes for qPCR

The identified reference genes were tested according to their stable expression in leaves to evaluate which genes are suitable for qPCR experiments. For this purpose, qPCR was performed with cDNA of three biological replicates of the empty vector control plants (pCottonA) and plants infiltrated with pCotton-PDS. The analysis of the most suitable reference genes was done with the tool NormFinder [25]. Used primers for each reference gene can be found in Additional file 1: Table S1.

### Plant cultivation

The cultivation of *Cannabis sativa* L. plants from individual seeds of the variety Finola was done under long day conditions (18 h light/6 h dark, light intensity 130 µM m^−2^ s^−1^, white light; 4000 K) at 25 °C on hydroculture. As fertilizer, a combination of FloraGro, FloraMicro and Flora Bloom was used (0.03% each, General Hydroponics Europe).The temperature was changed to 22 °C during the day and 18 °C in the night after infiltration with *A. tumefaciens*.

### Verification of predicted DNA sequences of *PDS* and *ChlI*

cDNA of *C. sativa* var. Finola was used to verify the predicted gene sequences of *PDS* and *ChlI.* The sequences were amplified with PCR and cloned into the vector pDionysos [26] by using the Gibson assembly method for better sequencing results. pDionysos was linearized with *Xba*I and for the amplification of *PDS* and *ChlI* the Q5® High-Fidelity polymerase (NEB) was used. Utilized primers can be found in Additional file 1: Table S3. After Gibson cloning, constructed plasmids were sequenced to verify the predicted gene sequences.

### Vector construction for agroinfiltration

For establishment of VIGS in *C. sativa* the *Cotton leaf crumple virus* (*CLCrV*) was applied. As a basis for the construction of the VIGS vectors the plasmids pJRT.Agro.CLCrVA.008 (pCottonA) (Addgene plasmid # 31809) carrying DNA-A and pJRT.Agro.CLCrVB1.3 (pCottonB) (Addgene plasmid # 37974) harboring DNA-B of the virus were used. Both plasmids were a gift from Niki Robertson [6].

Construction of the VIGS-vectors was done by linearizing the plasmid pCottonA with *Spe*I and cloning the selected 300 to 400 base pair fragments of *PDS* or *ChlI* into the mentioned linearized vector with the Gibson Assembly method. The resulting plasmids pCottonA-PDS and pCottonA-ChlI were sequenced to verify the correct assembly. All used primers are listed in Additional file 1: Table S3.

### Agroinfiltration of *C. sativa*

Competent *A. tumefaciens* LBA4404, GV3101 and AGL1 cells were transformed with the constructed plasmids according to a published protocol [27]. For agroinfiltration the transformed *A. tumefaciens* cells were cultivated in a 5 mL-preculture in YEB medium overnight at 28 °C for strains LBA4404 and GV3101 and 26 °C for AGL1 and 200 rpm with 50 mg L^−1^ kanamycin as well as respective antibiotics for each strain (Additional file 1: Table S2). The cultivation of the main cultures was done according to an existing protocol [28] with minor changes. For instance, cells from the preculture were inoculated in 30 mL YEB medium with 50 mg L^−1^ kanamycin and respective antibiotics for each strain as well as 10 mM MES and 20 µM acetosyringone. The cells were grown overnight at 26/28 °C and 200 rpm. For infiltration cells were harvested by centrifugation and washed twice in infiltration buffer (10 mM MES, 10 mM MgCl_2_, 200 µM acetosyringone). The cells were incubated at room temperature for 4 h in the dark, after the OD_600nm_ was adjusted to 4. After incubation, cells containing pCottonB were mixed in a ratio of 1:1 with cells containing DNA-A with the desired gene fragments of *PDS* or *ChlI.* The mixture was infiltrated into *C. sativa* var. Finola seedlings at the 4-leaf stage in the cotyledons and primary leaves with a needleless syringe. After infiltration the plants were cultivated at 22 °C in the light and 18 °C in the dark.

### cDNA synthesis and quantitative real-time PCR (qPCR)

Harvested plant leaves were immediately frozen in liquid nitrogen and total RNA was extracted (NucleoSpin RNA plus Kit; Macherey Nagel). For cDNA synthesis (LunaScript® RT Supermix Kit; NEB), 800 ng of DNase digested RNA were used. The cDNA was diluted to 5 ng µL^−1^ and qPCR was performed in five biological replicates with the Luna® Universal qPCR Master Mix (NEB) in 20 µL reactions according to manufacturer’s instructions. The following parameters were used for amplification: 95 °C denaturation for 60 s; 40 cycles of denaturation at 95 °C for 15 s and extension at 60 °C for 30 s. Afterwards, melting curves were measured. Calculation of C_t_ values was done with the StepOne V2.2.2 (Applied Biosystems) software. The data was analyzed with the use of the ∆∆C_t_-method [29]. Normalization of the samples was done by using the mean of the C_t_ values of *UBQ5* and *eIFa.* The expression level of *PDS* and *ChlI* were set to 1.0.

### Proof of virus infection

To test, if the virus is present in the plants, genomic DNA was isolated five weeks after infiltration (Nucleospin Plant II, Macherey–Nagel). Detection of Virus DNA-A and DNA-B was performed in PCR experiments with the use of virus gene specific primers, respectively. As negative control genomic DNA from wild type plants was used. Amplification was done using the Red Taq DNA Polymerase Mastermix (VWR). Primers can be found in Additional file 1: Table S3.

### Quantification of pigments

The extraction and quantification of photosynthetic pigments (chlorophyll a, chlorophyll b and carotenoids) was done according to an existing protocol [30] with minor changes. 50 mg of grounded, frozen leaf material was used for extraction of pigments with acetone. The acetone phase was filled up to 10 mL and 2 mL of the extract were filtered through a 0.45 µm nylon filter into a quartz cuvette. Photometric measurements were performed at 662, 645 and 470 nm.

## Results

### Identification of candidate genes and siRNAs prediction

**Fig. 2:**
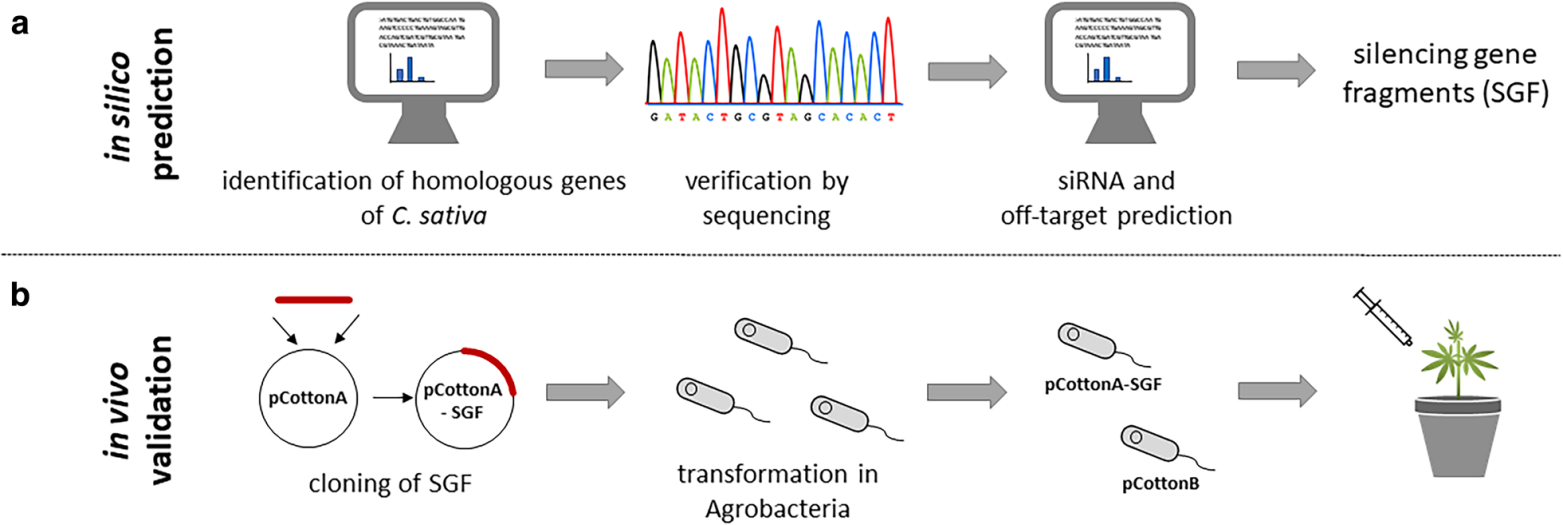
Workflow of a VIGS experiment **a** after identification of gene sequences and validation by sequencing, suitable siRNAs and off-targets are predicted. Based on these predictions silencing gene fragments were defined. **b** For in vivo validation silencing fragments are cloned in the VIGS-vectors and transformed in Agrobacteria for infiltration experiments

For the establishment of VIGS in *C. sativa,* it is favorable to use target genes, which lead to a visible phenotype, if their transcript level is downregulated. Suitable genes, already used in many other VIGS approaches, represent the genes encoding for the *phytoene desaturase* (*PDS*) and *magnesium chelatase subunit I* (*ChlI*). Both genes involved in the photosynthesis/carotenoid biosynthesis and silencing of these genes lead to white/yellow leaves phenotype. Since these genes were not identified from *C. sativa* yet, homologous sequences need to be identified from the cannabis draft genome and transcriptomic resources. For this purpose, reference sequences from *AtPDS* and *AtChlI of Arabidopsis thaliana* L. were used. To search for homologies, a command-line standalone BLAST+ for Linux (version 2.6.0+) was used. Afterwards, a database for the cannabis draft genome and the transcriptomic data was created by utilizing the “BLASTDB” algorithm, and with the use of “blastn”, respective homologous sequences were found (Fig. 2a). Sequences with at least 60% identity and a stringent e-value of 1e−05 were considered for further analysis. For verification of the candidate sequences, they were compared with the use of BLAST+ against NCBI non-redundant protein (nr), nucleotide (nt) and Swiss-Prot (UniProtKB) databases with the blastx algorithm. Only sequences with significant similarity of over 60% with the reference sequences of *ChlI* and *PDS* from other plant species were considered as potential homologs in *C. sativa*. Both identified sequences (*Cs-PDS* and *Cs-ChlI*) share over 80% homology at nucleotide and protein level compared to the reference sequences from Arabidopsis*.* Validation of the computationally predicted sequences was carried out by PCR amplification. Then the amplified coding sequences were cloned into a vector using Gibson assembly. Afterwards they were verified by Sanger sequencing. The results showed a significant similarity of 99.9% in comparison to the predicted sequences. Both sequences were submitted to NCBI (GenBank accession numbers: CsPDS, MN395698; CsChlI, MN395699) and can be found in Additional file 1.

To achieve efficient gene silencing it is necessary to select gene fragments that are able to form efficient siRNAs thereby possessing minimal off-target silencing effects. For this purpose, the identified sequences were analyzed by using the bioinformatics tool “pssRNAit” (https://plantgrn.noble.org/pssRNAit/). The tool predicted 21 siRNAs and 347 off-targets for *Cs-PDS* and 16 siRNAs and 259 off-targets for *Cs-ChlI*. To filter out off-target sites, which might have other binding sites other than *Cs-PDS* and *Cs-ChlI,* all found siRNAs were scanned against the cannabis database, described above. Finally, one siRNA was selected for each gene to design the silencing gene fragments (SGF). Fragments were considered, having a length of 150–400 base pairs and a GC content between 30 and 70%. The designed SGF for *PDS* had a length of 392 base pairs and the SGF chosen for *ChlI* had a fragment size of 300 base pairs.

### *Cotton leaf crumple virus* is suitable to induce PTGS in cannabis

We chose the *Cotton leaf crumple virus* (*CLCrV*), a circular ssDNA virus for VIGS experiments in *C. sativa*. At first, the selected *PDS* fragment was cloned into pCottonA for the generation of the plasmid pCotton-PDS, which was transformed into the three *A. tumefaciens* strains for evaluation of the best-performing strain. The DNA-B virus component (Fig. 1), located on the plasmid pCottonB and the empty DNA-A vector (pCottonA), were transformed into the same Agrobacterium strains. *C. sativa* plants were infiltrated either with pCotton-PDS or pCottonA as an empty-vector control in combination with pCottonB in a ratio of 1:1 (Fig. 2b). Plants were infiltrated with Agrobacteria at the 4-leaf stage with a needleless syringe, 7 to 10 days after germination into cotyledons and first leaves.

**Fig. 3:**
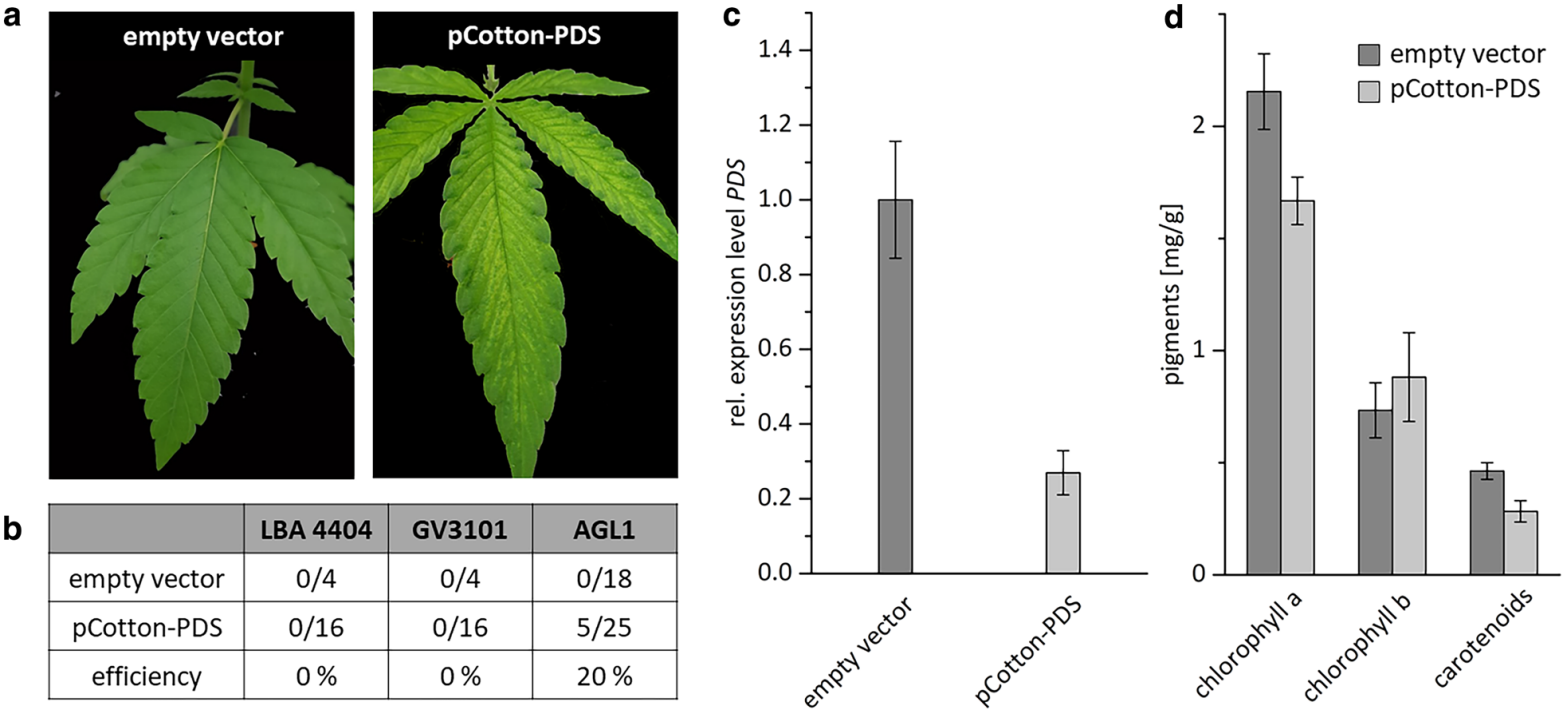
Silencing of *PDS* induced with the *Cotton leaf crumple virus* **a** Plants were infiltrated with the *A. tumefaciens* strain AGL1 carrying pCotton-PDS or an empty-vector control. Phenotypes could be identified approximately four weeks after infiltration with pCotton-PDS (right), whereas control plants showed no phenotype (left). **b** Overview on the efficiency of VIGS with different *A. tumefaciens* strains and number of infiltrated plants. **c** Quantification of the expression level of *PDS* in plants showing a phenotype and empty- vector control plants were analyzed by qPCR and normalized relative to the expression levels of *eIFa* and *UBQ5*. Error bars indicate standard error of five biological replicates. **d** Quantification of pigment content (chlorophyll a, chlorophyll b and carotenoids) of leaves from empty-vector control plants and *PDS-*silenced plants. Error bars indicate standard error of 3 representative replicates

In the beginning, 16 plants were infiltrated with each Agrobacterium strain. The infiltrated plants were grown in climate chambers and four weeks after infiltration leaves of two plants infiltrated with the strain AGL1 showed the desired phenotype of bleached leaves, indicated by small white spots (Fig. 3a). Control plants showed no phenotype. Notably, no phenotype could be observed for plants infiltrated with strains GV3101 and LBA4404 up to this point. Thus, it was suggested that using the strain AGL1 for infiltration of the plants is more effective with regard to gene silencing and further infiltrations were only carried out with the Agrobacterium strain AGL1. In total, 38 plants were infiltrated with the strain AGL1 carrying pCotton-PDS, whereof 13 plants died within 3 days. From the remaining 25 plants, five plants showed the desired phenotype on newly developed leaves, leading to an overall efficiency of 20% (Fig. 3b).

### Validation of the knockdown of *PDS* with qPCR

The white-dotted leaves of the pCotton-PDS-infiltrated plants indicate a downregulation of the transcript level of the *PDS* gene. However, no completely white leaves were observed. To investigate to which extend the transcript of *PDS* was downregulated in the described plants, quantification of the transcript level of *PDS* was done with quantitative real-time PCR (qPCR). For this purpose, it was necessary to find a suitable combination of reference genes. Nine reference genes could be identified from the cannabis transcriptomic data, which should be tested for stable expression either in control plants and plants infiltrated with pCotton-PDS, including the commonly used candidates *actin2*, *tubulin1*,* 18S rRNA*, *glyceraldehyde 3-phsophate dehydrogenase* (*GAPDH)*, *elongation factor 1α* (*EF1α*), *eukaryotic initiation factor a* (*eIFa*), *ubiquitin 5* and *ubiquitin* 10 (*ubq5*, *ubq10*). Additionally, the gene *yellow leaf specific protein 8* (*YLS8*) was considered, which was found to be a suitable reference gene in hop (*Humulus lupulus* L.) [31], a plant belonging to the Cannabaceae family as well. After isolation of RNA from leaves of three biological replicates of the empty vector control plants and plants infiltrated with pCotton-PDS, cDNA was synthesized and used for qPCR experiments. Each gene was analyzed in three technical replicates. To find the most stable reference gene, the tool NormFinder was used [25], which allows a prediction of the best reference gene or best combination of reference genes, by analyzing the C_T_ values of the samples. Most stable expression, i.e. lowest stability values were obtained for *UBQ5* and *eIFa* (Additional file 1: Table S4). Thus, they were used as reference genes for normalization in all following qPCR experiments.

In comparison to the control plants, infiltrated with the empty vector pCottonA, the transcript level of *PDS* was downregulated by 73% in plants (Fig. 3c), which were infiltrated with pCotton-PDS and showing a visible phenotype, as described above (Fig. 3a). This indicates, that the observed phenotype is linked to the downregulated transcript level and gene silencing of the *PDS* gene was possible with the predicted silencing fragment and the related predicted siRNA. Furthermore, this was confirmed by quantification of the pigments (chlorophyll a, chlorophyll b and carotenoids). Leaves with a visible phenotype showed a decrease of chlorophyll a and carotenoids by 27% and 29%, respectively. However, the chlorophyll b content was not affected (Fig. 3d).

### ChlI as a second marker for VIGS in cannabis

**Fig. 4:**
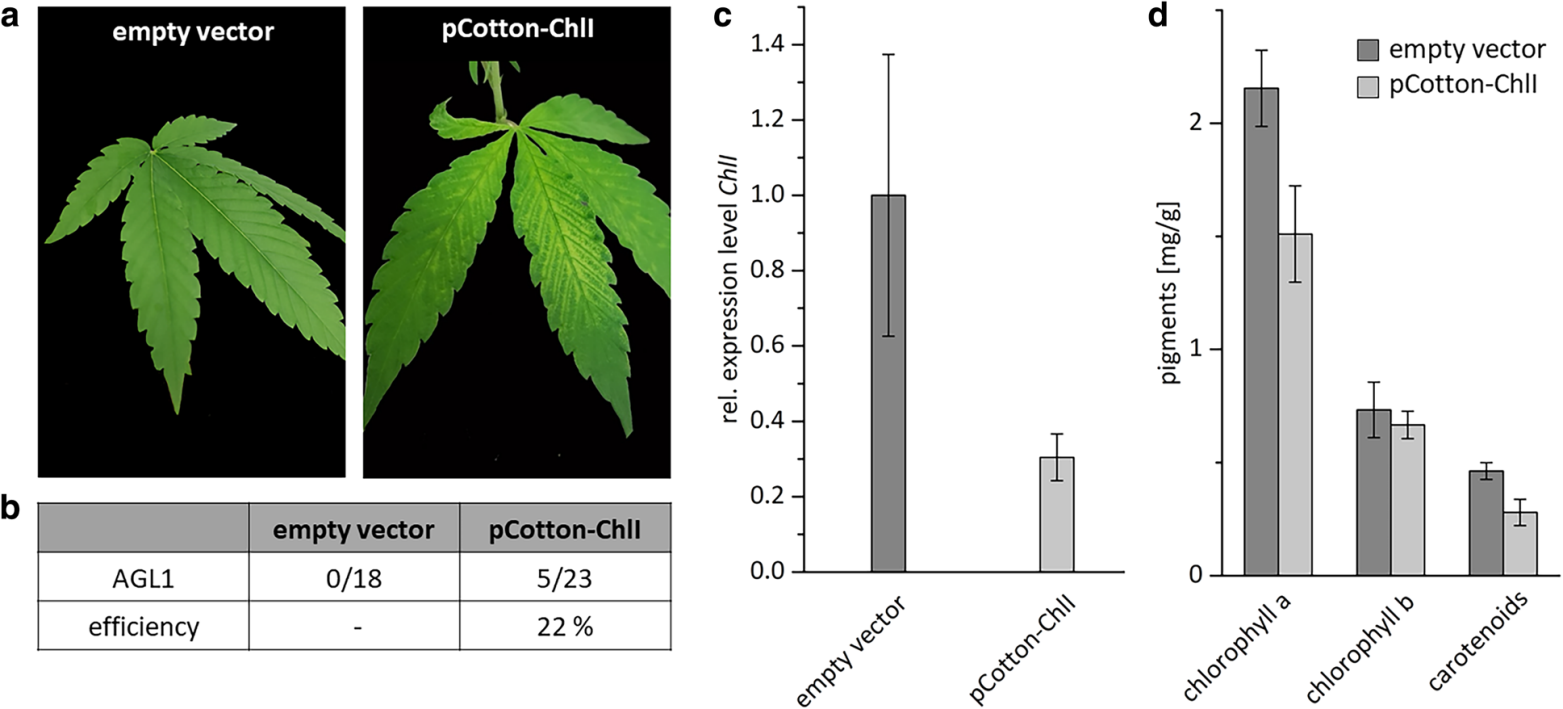
Silencing of *ChlI* induced with the *Cotton leaf crumple virus* **a** Plants were infiltrated with the *A. tumefaciens* strain AGL1 carrying pCotton-ChlI or an empty-vector control. Phenotypes were visible approximately four weeks after infiltration with pCotton-ChlI (right). No phenotype was visible in control plants (left). **b** Overview on the efficiency of VIGS if plants were infiltrated with the *A. tumefaciens* strain AGL1. **c** *E*xpression level of *ChlI* in plants showing a phenotype and empty-vector control plants were quantified with qPCR and normalized relative to the expression levels of *eIFa* and *UBQ5*. Error bars indicate standard error of five biological replicates. **d** Quantification of pigment content (chlorophyll a, chlorophyll b and carotenoids) of leaves from empty-vector control plants and *ChlI*-silenced plants. Error bars indicate standard error of 3 representative replicates

In a second approach, the developed system should be transferred to a second marker gene, showing a visible phenotype as well, the *ChlI* gene. Since this gene is related to photosynthesis, downregulation would lead to a bleaching phenotype as well. For cotton it was observed, that targeting *ChlI* for gene silencing had a greater visible bleaching effect than *PDS*-silencing [9]. To test this, the chosen silencing fragment was cloned into pCottonA and transformed only into *A. tumefaciens* AGL1 cells. As described above, cells were cultivated, harvested and the optical density was diluted to 4.0 in infiltration buffer. In total 40 plants were infiltrated with pCotton-ChlI, whereof 23 survived. As empty vector control, 8 plants were infiltrated with pCottonA. Whereas control plants showed no phenotype at all, five plants infiltrated with the silencing construct (pCotton-ChlI) showed the desired phenotype after approximately four weeks, representing an efficiency of 22%. As already observed for *PDS* silenced plants, the newly developed leaves are spotted with white dots all over the leaves. No complete bleaching was observed as well (Fig. 4a, b).

Transcript analysis of five biological replicates indicated a downregulation of 70% relative to the control plants infiltrated with the empty vector pCottonA (Fig. 4c). Similar to the PDS-silencing approach, the pigment content was analyzed to confirm the given results. Compared to the empty vector control plants chlorophyll a was decreased by 30% and the carotenoid content by 40% in *ChlI*-silenced plants.The chlorophyll b level was not affected (Fig. 4d).

### Virus is present in infiltrated plants

**Fig. 5:**
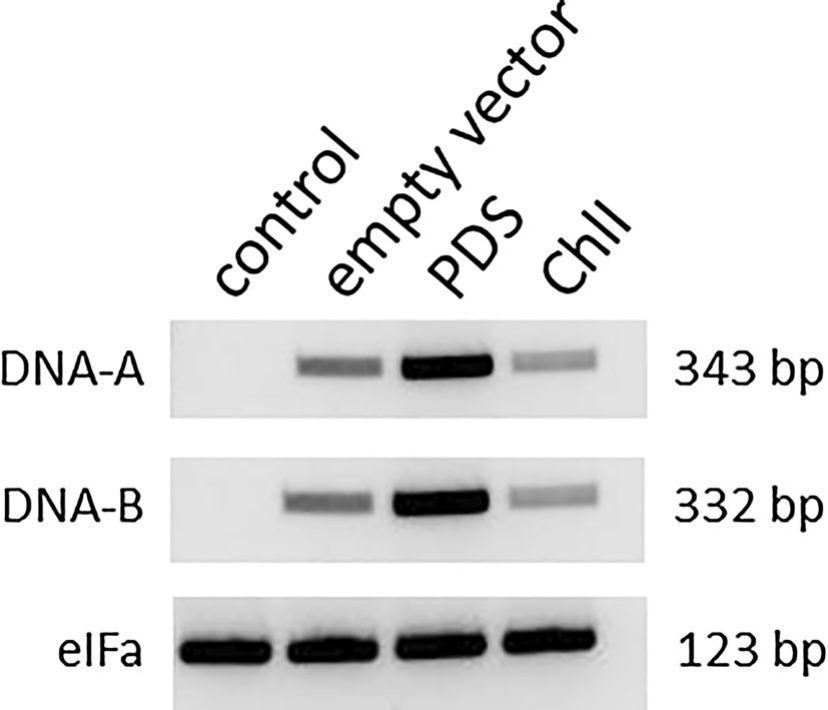
Detection of DNA-A and DNA-B. Genomic DNA was extracted either from *PDS*- and *ChlI*-silenced plants five week after inoculation or from control plants. As internal standard *eIFa* was used to ensure similar amounts of genomic DNA were used for amplification

Finally, the presence of both virus ssDNAs (DNA-A and DNA-B) was verified in agroinfiltrated plants. For this purpose primers specific for either DNA-A (AL1 gene) or DNA-B (BL1 gene) (Fig. 1) were designed amplifying short fragments. *eIFa* was used as internal control to ensure similar amounts of genomic DNA were used for amplification (Fig. 5). DNA-A and DNA-B fragments were present in plants transformed with empty vectors as well as in *PDS* and *ChlI* silenced plants, indicating both ssDNAs in all analyzed leaves.

### Discussion and conclusions

At the moment no reliable protocols are available, which enable regeneration and stable transformation of cannabis plants [4]. Nevertheless, having the ability to analyze individual gene functions of cannabis would be a great step to characterize the plant further. For this purpose, the method of virus-induced gene silencing was evaluated. Here we show for the first time that VIGS, can be applied to *C. sativa*, using the *Cotton leaf crumple virus*, for the downregulation of genes, by applying the virus to the plant cells transiently via agroinfiltration.

In first infiltration experiments with the *Cotton leaf crumple virus* and the three different *A. tumefaciens* strains LBA4404, GV3101 and AGL1, it could be shown, that only seedlings transformed with AGL1 showed the expected phenotype. This leads to the hypothesis that Agroinfiltration in *C. sativa* is only successful when using a hypervirulent *A. tumefaciens* strain, like AGL1 [32].

However, the observed phenotype of plants infiltrated with pCotton-PDS was not as strong as known from *tobacco rattle virus* (*TRV*)-infected plants. Only white and yellow spotted leaves instead of a complete bleaching of the green color was observed. Nearly the same phenotypes, i.e. white and yellow dots all over the surface of the leaves, were observed by applying this virus-system to the native host for gene knockdowns [6, 9]. Furthermore, the visible phenotype reflects the quantified pigment content, which was up to 40% lower compared to the empty-vector control plants.

To measure the expression levels of the targeted genes it was essential to set up a protocol for quantitative real-time PCR. For reliable results a combination of reference genes is required that shows the most stable expression. Nine potential reference genes were identified and analyzed with the NormFinder tool [25] resulting in *UBQ5* and *eIFa* as the best combination of reference genes for normalization. Since the coding sequences of potential reference genes are available by now, they can be used for designing future qPCR experiments e.g. for other tissues than leaves or different cannabis varieties, respectively.

According to the observed phenotype, it was not surprising that the silencing effect of 73% and 70% in our study, respectively, was not as high as in experiments utilizing the *TRV*. Interestingly, the comparison of our results with the silencing effects of other studies shows that the determined transcript levels are within the same range. In *N. benthamiana* and tomato gene silencing of minimum 78% was reached [8, 33]. With the use of the *Foxtail Mosaic Virus* in barley, transcription levels were reduced by minimum 75% [34].

In total, the silencing-efficiency of *PDS* and *ChlI* with 20% and 22%, respectively, is rather low. In cotton the silencing-efficiencies were 81% and 65% for agroinfiltration or particle bombardement, respectively [6]. However, in *N. benthamiana* plants silencing-efficiencies up to 98% could be reached by using *TRV* [35]. This demonstrates that *CLCrV* exhibits an overall lower silencing-efficiency compared to *TRV*. By using a non-native host, like cannabis, reduced efficiencies can be assumed. Nevertheless, so far no suitable cannabis-specific virus is known, which is able to show virus-induced symptoms and exhibit the capability to move between plant cells [36].

For tobacco and flax it could already be shown, that the removal of the apical shoot meristem, right after Agroinfiltration, led to a higher silencing efficiency, due to a more effective spread of the virus within the plant [37, 38]. However, it could be shown, that the virus is able to spread into new developing leaves, since fragments of both ssDNAs could be amplified. Since control plants showed no bleaching effect it can be concluded that the inserted fragments of *PDS* and *ChlI* were responsible for efficient gene silencing.

This shows, that despite the weak phenotype, the developed VIGS-method in *C. sativa* can be used to perform reverse genetic approaches to identify unknown gene functions. This will help to elucidate unknown biosynthetic routes and will contribute to a deeper knowledge of medical cannabis. Moreover, the established method is also suitable for the evaluation of endogenous miRNAs and their corresponding targets [39, 40]. This opens the possibility to elucidate gene functions and to decipher the regulatory network of altering transcription levels for a better understanding of the regulation of biosynthetic routes and plant development.

## Supplementary information

**Additional file 1. Table S1.** Primers used for qPCR experiments. **Table S2.** Antibiotic concentrations used for cultivation of Agrobacterium tumefaciens strains. **Table S3.** Primers used in the study for construction of the vectors. **Table S4.** Results from the NormFinder analysis of reference genes suitable for qPCR.

## Data Availability

The material used during the current study are available from the corresponding author on reasonable request.
