# Supplementary material for: Virus-induced gene silencing (VIGS) in Cannabis sativa L
Source: Plant Methods. 2019 Dec 26;15:157. doi: 10.1186/s13007-019-0542-5 (PMC6931244; doi:10.1186/s13007-019-0542-5)
Supplement: Supplementary file 1 — Additional file 1. Table S1. Primers used for qPCR experiments. Table S2. Antibiotic concentrations used for cultivation of Agrobacterium tumefaciens strains. Table S3. Primers used in the study for construction of the vectors. Table S4. Results from the NormFinder analysis of reference genes suitable for qPCR. [file 13007_2019_542_MOESM1_ESM.docx]

**Additional file**

**Virus-induced gene silencing (VIGS) in *Cannabis sativa*** l.

Julia Schachtsiek, Tajammul Hussain, Khadija Azzouhri Oliver Kayser and Felix Stehle*

*Laboratory of Technical Biochemistry, Department of Biochemical and Chemical Engineering, TU Dortmund University,Germany*

*Correspondence: Felix Stehle [felix.stehle@tu-dortmund.de](mailto:felix.stehle@tu-dortmund.de); phone +49 2317555115

**Coding sequences of *CsPDS* and *CsChlI***

>CsPDS

ATGTCTCAGTGGGGTTCTGTTTCAGCTACTAACTTCAGCTCCCACCAGAGTCGTATCTTT

GAATTTCCAAACGTAGGAACAGTACCCAGATGTTGTTTTTCATTGGGTTCCAAAAAAATG

GCGTCTTTGGCTTTTGGGTCTAATCAATTTATGCCTCCTGGTACAAGACTGAAGAAGAAG

AAGGGTGTTTTGCCTTTAAAGGTGGTTTGTGTGGATTATCCAAGACCAGAGCTTGATAGT

ACTGTTAACTTTTTGGAAGCTGCGGCTTTGTCTGCTTCTTTTCGTGGCTCTCCTCGTCCA

GCTAAGCCTTTGAAAGTTGTAATTGCTGGTGCAGGTTTGGCTGGTTTATCTACTGCAAAA

TACTTGGCAGATGCAGGTCATAAACCGATATTACTCGAAGCAAGAGATGTTTTAGGTGGA

AAGGTGGCAGCATGGAAAGATGATGACGGTGACTGGTATGAGACCGGTCTACATATATTC

TTTGGAGCTTATCCCAACATACAGAACTTGTTCGGAGAGCTAGGTATTGATGATCGGTTG

CAATGGAAAGAACATTCAATGATCTTTGCAATGCCCAGCAAGCCAGGAGAATTCAGCCGA

TTTGATTTCACCGATGCTCTGCCAGCTCCCTTAAATGGAATATGGGCCATTTTACGGAAC

AATGAGATGCTGACCTGGGCAGAAAAAGTCAAATTTGCAATTGGGCTTCTGCCTGCAATG

GTTGGCGGACAGCCTTATGTCGAAGCTCAAGATGGTTTCACCGTCAAAGATTGGATGAGA

AAGCAGGGCATACCTGATCGTGTAACGGATGAGGTCTTCATTGCCATGTCCAAGGCCTTA

AACTTCATTAACCCAGATGAACTTTCAATGCAATGTATATTGATTGCTTTAAACCGATTT

CTTCAGGAGAAGCATGGTTCCAAGATGGCTTTCCTAGATGGAAATCCCCCAGAGAGACTC

TGTATGCCCATAGTCGATCATATCCAGTCATTGGGCGGTGAAGTCCGACTTAATTCCCGT

ATACAAAAAATCGACCTGAATGATGATGGAACTGTAAAGAGATTTTTACTAACTAATGGT

AGTGAAATCGAAGGGGATGCATATGTGTTTGCAACACCAGTTGATATCCTCAAGCTTCTA

TTGCCCGAAAACTGGAAAGAGATTCCGTATTTCAAGAAATTGGATAAATTAGTTGGCGTC

CCCGTTATTAATGTCCACATATGGTTCGACCGAAAATTGAAGAACACATATGATCACCTT

CTTCTCAGCAGAAGTCCTCTCTTGAGTGTCTACGCCGATATGTCGGTAACTTGTAAGGAA

TATTACAATCCAAACCAGTCTATGCTAGAGTTGGTTTTCGCACCAGCAGAAGAATGGATT

TCGCGTAGTGACTCAGATATTATCGATGCTACGATGAAGGAGCTTTCTAAGCTCTTTCCT

GATGAAATAGCAGCTGATCAGAGCAAAGCGAAAATCTTAAAGTACCATGTTGTAAAAACA

CCGAGGTCTGTCTACAAAACTGTTCCTGATTGCGAACCTTGTCGCCCCAGACAAAGATCT

CCTCTTGAAGGTTTCTATTTAGCAGGTGACTACACAAAACAAAAATATTTGGCTTCAATG

GAAGGTGCGGTTTTGTCAGGAAAGTTTTGTGCACAAGCAATTGTACAGGATTATGAGTTG

CTTGCTGCTCGGGGTCAGAGAATTTTGGCCGAGGCTGGAAGTCGTTGA

>Cs_ChlI

ATGGCATCCGTCCTTGGAACTTCATCGTCAGCAATCTTGGCTTCTCGCTTCCTGTCTTCT

CCCTCCTCGAAGACTTCCAGTCCCTCTTTTTCTTTCAACCCAGGGCAGGGCAATGTGAAT

CGGTTGTATGGAGGAACCGGGATTCAGGTAAAAAAGGGGAGGTCTCAGTTCCACGTGGCA

GTTACCAATGTTGCTACTGAAATCAACTCTGTGGAACAGGACGGGAAGCTTAAAGCTAAG

AATAGTCAGAGGCCGGTTTACCCGTTTGCAGCCATAGTAGGACAAGATGAGATGAAACTT

TGTCTTCTCCTAAATGTGATTGACCCCAAGATTGGGGGTGTCATGATTATGGGTGATAGA

GGAACTGGAAAATCCACCACTGTAAGGTCTTTGGTTGATTTACTTCCCGAAATTAAGGTT

GTTTCTGGTGACCCTTACAACTCAGATCCAGAAGATCCAGAGTCTATGGGCGTGGAAGTC

AGAGAGAGTGTCTTGAAAGGGGACCAGCTTTCTGTTGTCTTGACTAAAATCAACATGGTT

GATTTACCTTTGGGTGCTACGGAAGATAGGGTATGCGGGACAATTGACATTGAGAAAGCT

CTGACTGAGGGTGTCAAAGCCTTTGAGCCTGGCCTTCTTGCAAAGGCCAACAGAGGAATT

CTATATGTAGATGAAGTAAATCTTTTGGATGATCACTTGGTTGATGTTTTATTGGATTCT

GCTGCTTCTGGTTGGAACACTGTGGAGAGAGAAGGTATTTCAATTTCACATCCTGCTCGG

TTTATTTTGATTGGCTCGGGAAATCCAGAAGAAGGGGAGCTCAGGCCACAACTTTTGGAC

CGTTTTGGAATGCATGCACAAGTGGGTACCGTAAGGGATGCAGAGCTTAGAGTGAAGATT

GTTGAGGAGAGATCTCGGTTTGATAAAAATCCCAACGAATTCCGCGAATCATATGACGCC

GAGCAAGAAAAACTGCAACAGCAAATTACTGCAGCTAGAAGTACTCTATCTTCTGTTCAG

ATCGACCATGAACTCAAAGTGAAAATTTCAAAGGTTTGTTCAGAGCTGAATGTTGATGGA

TTGAGAGGGGACATTGTAACAAACAGGGCTGCAAAAGCTTTGGCTGCTTTGAAGGGAAGA

GATAAAGTATCAACAGAGGATATTGCTACTGTCATCCCTAACTGCTTAAGACATCGTCTT

CGGAAGGATCCCTTGGAGTCAATTGATTCAGGTCTACTTGTCATTGAGAAATTTTATGAG

GTTTTCAACTGA

**Predicted siRNA sequences and silencing fragments:**

>PDS_siRNA

CAUAUGAUCACCUUCUUCU

>PDS fragment

ATTGAAGAACACATATGATCACCTTCTTCTCAGCAGAAGTCCTCTCTTGAGTGTCTACGC

CGATATGTCGGTAACTTGTAAGGAATATTACAATCCAAACCAGTCTATGCTAGAGTTGGT

TTTCGCACCAGCAGAAGAATGGATTTCGCGTAGTGACTCAGATATTATCGATGCTACGAT

GAAGGAGCTTTCTAAGCTCTTTCCTGATGAAATAGCAGCTGATCAGAGCAAAGCGAAAAT

CTTAAAGTACCATGTTGTAAAAACACCGAGGTCTGTCTACAAAACTGTTCCTGATTGCGA

ACCTTGTCGCCCCAGACAAAGATCTCCTCTTGAAGGTTTCTATTTAGCAGGTGACTACAC

AAAACAAAAATATTTGGCTTCAATGGAAGGT

>ChlI_siRNA

GUCUCCGGCCAAAUGGGCAAA

>ChlI-2

GGGCAATGTGAATCGGTTGTATGGAGGAACCGGGATTCAGGTAAAAAAGGGGAGGTCTCA

GTTCCACGTGGCAGTTACCAATGTTGCTACTGAAATCAACTCTGTGGAACAGGACGGGAA

GCTTAAAGCTAAGAATAGTCAGAGGCCGGTTTACCCGTTTGCAGCCATAGTAGGACAAGA

TGAGATGAAACTTTGTCTTCTCCTAAATGTGATTGACCCCAAGATTGGGGGTGTCATGAT

TATGGGTGATAGAGGAACTGGAAAATCCACCACTGTAAGGTCTTTGGTTGATTTACTTCC

**Predicted sequences of reference genes**

>Cs_actin

ATGGCCGACGCAGAGGACATTCAGCCCCTCGTTTGTGACAATGGTACTGGAATGGTGAAGGCTGGGTTTGCTGGCGATGATGCGCCCAGGGCAGTCTTTCCCAGTATTGTTGGTCGTCCTAGACACACTGGTGTTATGGTTGGAATGGGACAGAAGGACGCATACGTAGGTGATGAAGCCCAGTCTAAAAGAGGTATCCTTACTTTGAAATATCCCATCGAGCATGGTATTGTTAGCAACTGGGATGATATGGAGAAAATTTGGCATCATACTTTCTACAATGAACTTCGTGTTGCCCCAGAAGAGCACCCAGTGCTTCTCACTGAGGCTCCTTTGAATCCTAAGGCTAACAGAGAAAAGATGACCCAGATCATGTTTGAGACATTCAATGTTCCAGCTATGTATGTGGCCATCCAGGCAGTTTTGTCCCTTTACGCTAGTGGACGTACAACTGGTATCGTTCTGGATTCTGGTGATGGTGTGAGTCACACTGTGCCAATCTACGAAGGTTATGCCCTTCCCCATGCCATTCTTCGTCTTGACCTTGCTGGGCGTGATCTCACTGATTCTTTGATGAAGATTCTCACTGAGAGAGGCTACATGTTCACCACCACTGCCGAACGGGAAATTGTCCGTGACATGAAGGAGAAGCTTGCTTATGTTGCCTTGGACTATGAGCAGGAACTCGAGACTGCCAAGAGCAGCTCCTCAGTTGAGAAGAACTACGAGTTGCCCGATGGCCAGATTATCACCATTGGAGCTGAGAGATTCCGATGCCCAGAAGTCCTCTTCCAACCATCCCTCATTGGAATGGAAGCTGCAGGAATTCACGAGACCACCTACAATTCCATCATGAAGTGTGATGTGGATATCAGAAAGGATCTCTACGGTAACATTGTTCTCAGTGGTGGTTCTACTATGTTCCCTGGTATTGCAGACAGGATGAGCAAGGAGATCACAGCTCTTGCACCAAGCAGCATGAAGATTAAGGTTGTGGCTCCACCGGAGAGAAAATACAGTGTCTGGATCGGAGGGTCCATTCTTGCATCCCTCAGCACCTTCCAGCAGATGTGGATTTCCAAGGGCGAATACGATGAGTCAGGTCCATCCATTGTCCACAGGAAGTGCTTCTAA

>Cs_beta tubulin (partial)

ATGAGGGAAATCTTGCATATTCAAGGAGGTCAATGTGGTAACCAAATCGGTTCCAAGTTCTGGGAAGTAATCTGTAACGAGCATGGTGTAGACCCTACCGGGAGGTACCAGAGCGATGGTGCTGCTGATCTTCAGTTGGAGAGGATTAATGTCTACTACAATGAGGCTTCTGGAGGAAGGTACGTTCCTCGGGCTGTTCTTATGGATCTCGAACCTGGAACTATGGATAGCATCAGATCGGGTCCTTATGGACAGATCTTTCGCCCTGATAACTTCGTTTTCGGCCAGTCCGGTGCTGGAAACAATTGGGCCAAAGGTCACTACACCGAAGGCGCTGAGTTGATCGATTCGGTACTTGATGTTGTTCGTAAAGAGGCTGAAAACTGTGACTGTCTTCAAGGTTTTCAGGTATGTCACTCACTTGGAGGAGGGACTGGTTCTGGTATGGGAACACTTCTCATATCAAAGATCAGAGAGGAATATCCAGACAGAATGATGCTCACATTCTCAGTTTTCCCTTCTCCAAAGGTCTCTGACACAGTTGTGGAACCATACAATGCCACCCTCTCGGTGCATCAACTGGTTGAAAACGCTGATGAGTGCATGGTTCTTGATAATGAAGCACTTTATGATATTTGCTTCAGGACTCTAAAACTCAGCACACCAAGTTTTGGCGACTTGAACCATTTGATATCTGCAACTATGAGTGGTGTAACTTGCTGCCTGAGGTTCCCTGGGCAACTCAACTCGGACCTTCGTAAGCTGGCTGTTAATTTGATTCCATTCCCGCGACTTCACTTCTTCATGGTGGGGTTTGCACCTCTGACTTCTCGTGGATCCCAACAGTACATCTCCCTCACTGTGCCAGAGCTTACTCAGCAAATGTGGGATGCCAAGAACATGATGTGCGCAGCTGACCCTCGCCATGGCCGATACCTGACTGCCTCGGCTATGTTCAGGGGTAAGATGAGTACTAAAGAGGTGGATGAACAGATGATCAATGTGCAAAATAAGAACTCATCTTACTTTGTTGAGTGGATCCCAAACAACGTGAAATCAAGTGTTTGTGATATTCCACCATTGGGGCTTAAAATGGCGTCTACCTTTGTTGGTAACTCAACGTCGATCCAGGAGATGTTCAGGAGGGTGAGCGAGCAGTTCACAGCTATGTTCCGTCGCAAGGCCTTTTTGCATTGGTACACAGGAGAAGGAATGGACGAGATGGAGTTCACAGAGGCAGAGAGTAACATGAATGATTTGGTCGCTGAGTATCAACAGTACCAGGACGCCACTGCTGATGAGGAAGGTGAATATGAAGAAGAAGAAGAA

>Cs_18S_rRNA (partial)

TACCTGGTTGATCCTGCCAGTAGTCATATGCTTGTCTCAAAGATTAAGCCATGCATGTGT

AAGTATGAACTAATTCAGACTGTGAAACTGCGAATGGCTCATTAAATCAGTTATAGTTTG

TTTGATGGTATCTGCTACTCGGATAACCGTAGTAATTCTAGAGCTAATACGTGCAACAAA

CCCCGACTTCTGGAAGGGATGCATTTATTAGATAAAAGGTCGACGCGGGCTCTGCCCGTT

GCTCTGATGATTCATGATAACTCGACGGATCGCACGGCCTTCGTGCCGGCGACGCATCAT

TCAAATTTCTGCCCTATCAACTTTCGATGGTAGGATAGTGGCCTACTATGGTGGTGACGG

GTGACGGAGAATTAGGGTTCGATTCCGGAGAGGGAGCCTGAGAAACGGCTACCACATCCA

AGGAAGGCAGCAGGCGCGCAAATTACCCAATCCTGACACGGGGAGGTAGTGACAATAAAT

AACAATACCGGGCTCTACGAGTCTGGTAATTGGAATGAGTACAATCTAAATCCCTTAACG

AGGATCCATTGGAGGGCAAGTCTGGTGCCAGCAGCCGCGGTAATTCCAGCTCCAATAGCG

TATATTTAAGTTGTTGCAGTTAAAAAGCTCGTAGTTGGACCTTGGGTTGGGTCGATCGGT

CCGCCTCCGGTGTGCACCGGTCGGCTCGTCCCTTCTACCGGCGATGCGCTCCTGGCCTTA

ATTGGCCGGGTCGTGCCTCCGGTGCTGTTACTTTGAAGAAATTAGAGTGCTCAAAGCAAG

CCTACGCTCTGTATACATTAGCATGGGATAACATCATAGGATTTCGGTCCTATTCTGTTG

GCCTTCGGGATCGGAGTAATGATTAACAGGGACAGTCGGGGGCATTCGTATTTCATAGTC

AGAGGTGAAATTCTTGGATTTATGAAAGACGAACAACTGCGAAAGCATTTGCCAAGGATG

TTTTCATTAATCAAGAACGAAAGTTGGGGGCTCGAAGACGATCAGATACCGTCCTAGTCT

CAACCATAAACGATGCCGACCAGGGATTGGCGGATGTTGCTTTTAGGACTCCGCCAGCAC

CTTATGAGAAATCAAAGTTTTTGGGTTCCGGGGGGAGTATGGTCGCAAGGCTGAAACTTA

AAGGAATTGACGGAAGGGCACCACCAGGAGTGGAGCCTGCGGCTTAATTTGACCCAACAC

GGGGAAACTTACCAGGTCCAGACATAGTAAGGATTGACAGATTGAGAGCTCTTTCTTGAT

TCTATGGGTGGTGGTGCATGGCCGTTCTTAGTTGGTGGAGCGATTTGTCTGGTTAATTCC

GTTAACGAACGAGACCTCAGCCTGCTAACTAGCTATGCGGAGG

>Cs_GAPDH (partial)

GACAAGAAGATCAAGATCGGAATCAACGGTTTCGGAAGAATTGGACGTTTGGTTGCTAGA

GTTGCTCTCCAGAGGGACGATGTTGAGCTTGTCGCTGTTAACGATCCATTTATCACCACT

GATTACATGACATACATGTTTAAGTACGATTCCGTTCATGGACCATGGAAGCATCATGAG

CTTAAGGTCAAGGACTCTAAGACCCTTCTCTTCGGTGAGAAGCCCGTCACTGTTTTCGGT

GTCAGGAACCCAGAAGAAATCCCATGGGGCGAGACCGGTGCCGACTTTGTTGTTGAATCA

ACTGGAGTTTTCACTGACAAGGACAAAGCTGCTGCTCACTTGAAGGGTGGTGCTAAGAAG

GTTATCATCTCTGCCCCAAGTAAAGATGCACCCATGTTCGTTGTTGGTGTCAATGAGAAC

GAATACAAGCCAGAGTACGATATTGTTTCCAATGCTAGTTGCACTACCAATTGCCTTGCC

CCATTGGCCAAGGTTATCAACGACAGGTTTGGAATTGTTGAGGGTTTGATGACCACCGTC

CACTCCATCACTGCTACCCAGAAAACTGTTGATGGACCATCAAGCAAGGACTGGAGAGGC

GGAAGAGCCGCTTCCTTCAACATCATTCCCAGCAGCACTGGAGCTGCTAAGGCTGTTGGA

AAGGTGCTCCCAGCTCTTAATGGTAAATTGACCGGTATGTCTTTCCGTGTTCCTACCGTC

GATGTCTCAGTTGTTGACCTCACCGTCAGGCTTGCAAAGGCTGCAACCTACGACGACATC

AAAAATGCCATCAAGGAGGAGTCTGAGGGCAAATTGAAGGGTATCTTGGGATACACCGAA

GATGATGTTGTCTCTACCGACTTCATTGGTGACAGCAGGTCAAGCATCTTCGATGCCAAG

GCCGGAATTGCTTTGAACGAGAACTTTGTGAAGCTTGTGTCGTGGTACGACAACGAATGG

GGTTACAGTTCCCGTGTTGTTGACTTGATTGTCCACAT

>Cs_Ef1a

CACATCAACATCGTGGTCATCGGCCATGTCGACTCCGGCAAGTCGACCACCACTGGTCAC

CTTATCTATAAGCTTGGAGGAATTGACAAGCGTGTGATTGAGAGGTTTGAGAAGGAAGCT

GCTGAGATGAACAAGAGGTCATTCAAGTATGCTTGGGTTCTTGACAAGCTTAAGGCTGAG

CGTGAGCGTGGTATCACCATTGACATTGCCCTGTGGAAGTTTGAGACCACCAAGTACTAC

TGCACAGTCATTGATGCTCCTGGCCATCGTGACTTTATCAAGAACATGATTACTGGTACT

TCACAGGCTGATTGTGCTGTTCTCATCATTGATTCCACCACTGGTGGTTTTGAAGCTGGT

ATCTCTAAGGATGGACAGACCCGTGAGCATGCTCTTCTTGCTTTCACCCTTGGTGTCAGG

CAGATGATCTGTTGTTGTAACAAGATGGATGCCACCACACCCAAATACTCCAAGGCCAGG

TATGAGGAAATCGTGAAGGAAGTCTCTTCCTACTTGAAAAAGGTTGGGTACAACCCTGAC

AAAATCCCATTCGTTCCAATCTCTGGTTTCGAAGGAGACAACATGATTGAGAGGTCTACC

AACCTTGACTGGTACAAGGGTCCAACTCTTCTTGAAGCTCTTGACAATCTCTCTGAGCCC

AAGAGACCCTCAGACAAGCCACTCCGTCTTCCACTTCAGGATGTCTACAAGATTGGTGGT

ATTGGAACTGTGCCAGTGGGTCGTGTTGAGACTGGTGTCATCAAGCCTGGTATGGTTGTC

ACCTTTGCTCCCACCGGTCTGACCACTGAGGTCAAGTCAGTTGAGATGCACCACGAGGCT

CTTCTCGAGGCTCTTCCCGGTGACAATGTTGGGTTCAATGTTAAGAATGTGGCAGTTAAG

GATCTCAAGCGTGGTTACGTTGCATCCAACTCCAAGGATGATCCTGCCAAGGAGGCAGCC

AACTTCACTGCTCAGGTCATTATCATGAACCACCCCGGTCAAATTGGCAACGGCTATGCC

CCAGTTCTCGACTGCCACACCTCCCACATTGCTGTCAAGTTTTCTGAAATCCTAACCAAG

ATTGACCGTAGGTCTGGTAAGGAGCTCGAGAAGGAACCCAAGTTCTTGAAGAATGGTGAT

GCAGGTATGGTTAAGATGGTTCCAACCAAGCCCATGGTGGTTGAAACCTTCGCTGAGTAC

CCACCACTCGGACGTTTTGCTGTCCGTGACATGCGTCAAACCGTTGCTGTTGGTGTCATC

AAGAGTGTGGAGAAGAAGGACCCATCAGGAGCTAAGGTGACCAAGTCTGCTGCCAAGAAG

AAGTGA

>Cs_eIFa (partial)

ATGGCAGGTTTAGCACCAGAAGGCTCTCAATTTGATGCTCGTCAGTATGATTCCAAGATG

AGTGAATTACTGGAAAGTGATGGAAAAGATTTCTTTACATCATATGATGAGGTTCATGAA

AGTTTTGATGATATGGGTTTGCAAGAAAATCTTCTAAGAGGCATTTATGCATATGGTTTT

GAGAAGCCATCTGCAATTCAGCAGAGAGGGATTGTCCCCTTTTGTAAAGGACTTGATGTG

ATTCAACAAGCACAGTCTGGAACTGGAAAGACTGCTACTTTCTGCTCTGGAATTCTGCAG

CAGCTTGACTATGGCTTACTTGATTGCCAGGCTTTGGTTCTTGCACCCACTCGAGAACTT

GCCCAACAGATCGAGAAGGTTATGAGGGCTCTTGGTGATTATCTCGGTGTGAAGGTTCAT

GCATGTGTTGGTGGTACCAGTGTCCGTGAAGATCAACGTATTCTTTCCAGTGGAGTGCAT

GTTGTTGTTGGTACCCCTGGTCGTGTGTTTGACATGCTTCGGAGACAATCCCTGCGTCCT

GATAACATCAAAATGTTTGTTTTGGATGAAGCTGATGAAATGCTTTCTAGGGGTTTCAAG

GATCAGATTTATGACATTTTTCAATTGCTACCGCCAAAGATTCAGGTCGGTGTGTTCTCT

GCTACAATGCCACCTGAGGCTCTTGAGATCACCAGGAAGTTCATGAACAAACCTGTGAGA

ATCTTGGTGAAACGTGATGAGCTCACCCTCGAGGGTATCAAACAATTTCACGTCAACGTG

GAGAAAGAGGAGTGGAAGCTCGAGACACTTTGTGATCTTTACGAAACATTGGCAATCACC

CAGAGTGTTATCTTTGTTAACACAAGGCGAAAGGTCGATTGGCTCACAGACAAGATGCGC

AGCAGAGATCACACAGTTTCCGCTACCCACGGAGACATGGACCAGAACACTAGGGACATC

ATCATGAGGGAATTCCGATCAGGTTCCTCTAGAGTCCTCATCACCACCGATCTCTTAGCT

CGTGGTATTGATGTGCAACAGGTTTCTCTTGTTATAAACTTTGATCTTCCAACACAGCCC

GAAAACTACCTCCATCGTATCGGTCGTAGTGGACGATTCGGGAGAAAGGGTGTTGCCATT

AACTTTGTGACCAAAGATGATGAAAGAATGCTGTTCGATATTCAGAAGTTTTACAATGTG

GTTGTCGAAGAGCTGCCTGCAAATGTTGCTGA

>Cs_UBQ5 (partial)

ATGCAGATCTTCGTGAAAACCCTAACGGGAAGACTATAACCCTAGAGGTTGAGTCTTCCG

ATACCATCGACAATGTCAAGGCGAAGATCCAGGACAAGGAGGGCATCCCACCGGACCAGC

AGCGTCTCATCTTCGCCGGGAAGCAGCTAGAGGACGGCCGAACCCTAGCCGATTACAACA

TCCAGAAGGAGTCGACTCTTCACTTGGTGCTCAGACTTAGGGGAGGAGCCAAAAAGAGGA

AGAAGAAGACCTACACCAAGCCAAAGAAGATCAAACATAAGAAGAAGAAGGTGAAGCTCG

CTCTTCTCCAGTTCTATAAGGTTGATGACTCCGGTAAGGTGCAGCGGCTGCGGAAGGAGT

GTCCCAACGCTGAGTGCGGCGCCGGAACTTTCATGGCTAACCACTTCGACCGACATTACT

GCGGCAAGTGTGGTTTGACCTATGTGTATCAGAA

>Cs_UBQ10 (partial)

ATCCAAGACAAGGAGGGTATCCCACCAGACCAGCAGAGACTTATCTTTGCCGGTAAGCAA

CTCGAGGATGGGAGGACTCTTGCTGATTACAATATTCAAAAGGAGTCTACTCTCCACTTG

GTTCTCCGTCTTCGTGGTGGTATGCAGATTTTTGTAAAGACCCTTACAGGAAAGACAATA

ACCTTGGAGGTTGAGAGCTCCGACACTATTGACAATGTCAAAGCAAAGATCCAAGACAAG

GAAGGTATCCCACCAGATCAGCAGAGACTTATCTTTGCCGGTAAGCAACTCGAGGATGGA

AGGACACTTGCTGACTACAACATTCAGAAGGAGTCCACACTTCATCTTGTGCTTCGTCTT

CGTGGCGGTATGCAAATATTTGTGAAGACCCTTACAGGAAAGACCATCACCCTTGAGGTA

GAAAGCTCGGATACAATTGACAATGTAAAGGCGAAAATTCAAGACAAGGAAGGAATCCCA

CCAGACCAGCAAAGACTTATCTTTGCCGGTAAGCAATTGGAAGATGGCAGGACTTTAGCT

GATTACAACATCCAAAAGGAGTCGACCCTTCATTTGGTGCTGCGTCTGAGGGGTGGCATG

CAGATCTTTGTGAAGACCTTAACTGGAAAGACCATCACTTTGGAGGTAGAAAGCTCGGAT

ACAATAGACAATGTAAAGGCGAAAATTCAGGACAAAGAGGGCATCCCACCAGACCAGCAA

AGACTTATCTTCGCCGGTAAGCAACTCGAGGATGGAAGGACTCTTGCAGATTACAACATC

CAGAAGGAGTCTACTCTTCACCTCGTGTTGCGTCTGAGGGGTGGTATGCAGATCTTTGTT

AAGACCTTGACCGGAAAGACCATAACTTTGGAGGTGGAAAGTTCGGATACCATAGACAAT

GTAAAGGCGAAAATTCAGGACAAAGAGGGTATCCCACCAGACCAGCAGAGGTTGATTTTT

GCTGGTAAACAGTTGGAAGATGGTAGGACTTTGGCTGATTACAACATTCAGAAAGGAGTC

TACTCTTCACCTTGTTCTCCGTCTTCGTGGCG

>Cs_YLS8

ATGTCGTACTTGCTTCCACATTTGCACTCTGGATGGGCCGTAGATCAGGCCATCCTCGCC

GAGGAAGAGCGTCTCGTCATCATCCGATTTGGCCACGACTGGGATGAGACCTGTATGCAG

ATGGATGAAGTTTTGTCATCAGTTGCTGAGACAATCAAAAACTTTGCAGTGATATACCTT

GTCGACATCACTGAGGTTCCAGATTTCAACACAATGTACGAGTTGTATGACCCATCTACG

GTCATGTTTTTCTTCAGGAACAAGCACATTATGATCGATCTCGGAACTGGAAACAATAAT

AAGATCAACTGGGCCCTCAAGGATAAGCAAGAGTTCATTGACATTGTTGAGACTGTGTAC

CGTGGAGCAAGGAAGGGACGGGGTCTTGTGATTGCTCCTAAGGATTACTCTACCAAATAT

CGCTACTAA

**Table S1: Primers used for qPCR experiments**

| **Primer name** | **Sequence 5’ to 3’** |
| --- | --- |
| ACT2_fwd | CTGCCGAACGGGAAATTGTC |
| ACT2_rev | AACTGAGGAGCTGCTCTTGG |
| TUB1_fwd | TTCCATTCCCGCGACTTCAC |
| TUB1_rev | GCGCACATCATGTTCTTGGC |
| 18S_fwd | ACCATAAACGATGCCGACCAG |
| 18S_fwd | TTCAGCCTTGCGACCATACTC |
| GAPDH_fwd | TGTCTTTCCGTGTTCCTACC |
| GAPDH_rev | TCAATTTGCCCTCAGACTCC |
| EF1a_fwd | AGCGTGGTATCACCATTGAC |
| EF1a_rev | AGCACAATCAGCCTGTGAAG |
| eIF4a_fwd | CAATCCCTGCGTCCTGATAAC |
| eIF4a_fwd | GACCTGAATCTTTGGCGGTAG |
| UBQ5_fwd | AAGCTCGCTCTTCTCCAGTTC |
| UBQ5_rev | CACACTTGCCGCAGTAATGTC |
| UBQ10_fwd | GGAAGGTATCCCACCAGATCAG |
| UBQ10_rev | CCACGAAGACGAAGCACAAG |
| YLS8_fwd | CTCGTCATCATCCGATTTGG |
| YLS8_rev | CCGTAGATGGGTCATACAACTC |
| PDS_fwd | CCAAGACCAGAGCTTGATAG |
| PDS_rev | ACCTGCACCAGCAATTAC |
| ChlI_fwd | CTTGGCTTCTCGCTTCCTGTC |
| ChlI_rev | TACCTGAATCCCGGTTCCTCC |

**Table S2: Antibiotic concentrations used for cultivation of *Agrobacterium tumefaciens* strains**

| 1. **tumefaciens strain** | **Antibiotics** |
| --- | --- |
| GV3101 | 50 μg mL^-1^ gentamycin,  25 μg mL^-1^ rifampicin |
| LBA4404 | 50 μg mL^-1^ streptomycin  25 μg mL^-1^ rifampicin |
| AGL1 | 100 µg mL^-1^ carbenicillin |

**Table S3: Primers used in the study for construction of the vectors**

| **Primer Name** | **Sequence 5’ to 3’** | **Purpose** |
| --- | --- | --- |
| pDio_PDS_fw | TCACACTGGCGGCCGCTCGAGCATGCATATGTCTCAGTGGGGTTCTGTT | validation of *PDS* |
| pDio_PDS_rv | ATAACTAATTACATGATGCGGCCCTTCAACGACTTCCAGCCTCGG |  |
| pDio_ChlI_fw | TCACACTGGCGGCCGCTCGAGCATGCATATGGCATCCGTCCTTGGAA | validation of *ChlI* |
| pDio_ChlI_rv | ATAACTAATTACATGATGCGGCCCTTCAGTTGAAAACCTCATAAAATTTCTC |  |
| pTRV-PDS_fw | TGACTACCATGGATTGAAGAACACATATGATCACCTTCTTC | construction pTRV2-PDS |
| pTRV-PDS_rv | ACGCATCTCGAGACCTTCCATTGAAGCCAAATA |  |
| PCR_TRV_fw | CGGACGAGTGGACTTAGATTC | colony PCR |
| PCR_TRV_rv | CGATCAATCAAGATCAGTCGAGAATG |  |
| Gib-PDS1_fw | TGGCCAGTTAACGCTAGCGAATTCAATTGAAGAACACATATGATCAC | Gibson pCotton-PDS |
| Gib-PDS1_rv | TAGAGCAAAATGGCATGCCTGCAGAACCTTCCATTGAAGCCAAA |  |
| Gib-ChlI2_fw | TGGCCAGTTAACGCTAGCGAATTCAGGGCAATGTGAATCGGTTGT | Gibson pCotton-ChlI |
| Gib-ChlI2_rv | TAGAGCAAAATGGCATGCCTGCAGAGGAAGTAAATCAACCAAAGACCTTACA |  |
| SS129 | CACAGGAAACAGCTATGAC | colony PCR |
| PCR_VIGS_rv | ACGTCCAGATCCGATTCAAC | colony PCR |
| pCottonA_fw | GAACTGGTTTCCCGTACTTG | presence of virus |
| pCottonB_rv | GCCGTATGTAGTAACGATCC | presence of virus |
| pCottonB_fw | TACAATGGCCCACAACAC | presence of virus |
| pCottonA_rv | GTCCCTCCATTTCCACTTTC | presence of virus |

**Table S4:** Results from the NormFinder analysis of reference genes suitable for qPCR. Analysis was done with three biological replicates.

| **Gene name** | **Stability value** |  | **Best gene** | eIFa |
| --- | --- | --- | --- | --- |
| ACT2 | 0.256 |  | **Stability value** | 0.169 |
| EF1a | 0.739 |  |  |  |
| eIFa | 0.169 |  | **Best combination of two genes** | eIFa and UBQ5 |
| GAPDH | 0.359 |  | **Stability value for best combination of two genes** | 0.144 |
| TUB1 | 1.077 |  |  |  |
| UBQ5 | 0.233 |  |  |  |
| UBQ10 | 0.517 |  |  |  |
| YLS8 | 0.368 |  |  |  |
| 18 S | 0.230 |  |  |  |
